# Causes behind insect folivory patterns in latitudinal gradients

**DOI:** 10.1111/j.1365-2745.2010.01707.x

**Published:** 2011-03

**Authors:** Christer Björkman, Åsa Berggren, Helena Bylund

**Affiliations:** 1 Department of Ecology, Swedish University of Agricultural SciencesPO Box 7044, SE-750 07 Uppsala, Sweden; 2 Swedish Species Information Centre, Swedish University of Agricultural SciencesPO Box 7007, SE-750 07 Uppsala, Sweden

**Keywords:** climate, herbivory, plant–herbivore interactions, temperature, trophic interactions

## Abstract

**1.**Adams and Zhang recently published one of the best studies so far of patterns of insect folivory along a latitudinal (climatic) gradient. They show clear negative trends in foliage loss in relation to temperature for certain groups of insect herbivores.

**2.**Although their suggestion that the plant–herbivore interaction may be more important in cooler climates could be valid, they did not bring up the complementary explanation that interactions between predators and herbivores could also vary with climate. There are indications that insect natural enemies may respond more positively than insect herbivores to an increase in temperature. We argue that higher predator pressure in warmer climates may partly explain the patterns observed by Adams and Zhang.

**3.***Synthesis*.To further develop the important research concerning herbivory in a changing climate, both theoretically and empirically, plant ecologists and entomologists would mutually benefit from joining forces.

The hypothesis that herbivore damage should be higher in warmer climates (e.g. Coley & Barone 1996), predicting that climate warming should lead to more herbivory at a given latitude (Wolf, Kozlov & Callaghan 2008), was recently questioned by Adams & Zhang (2009). The authors made a survey of levels of insect folivory on a subset of common tree species over a latitudinal gradient along the North American east coast. The results clearly showed negative relationships between foliage loss (%) and temperature; the *r*-values, including trends with *P*-values < 0.07, ranged between −0.243 and −0.695. Similar results have been reported elsewhere (e.g. Lowman 1984), but there are examples showing opposite trends (Coley & Barone 1996) and the most common observation seems to be no trends (e.g. Andrew & Hughes 2005; Sinclair & Hughes 2008). The study by Adams & Zhang (2009) is one of the best of its kind, partly because the authors distinguish between damage caused by different types of insect herbivores, which has not been done in any of the previous studies. In addition, the study was repeated in time over 2 years. Still, Adams & Zhang (2009) neglects one important aspect, i.e. the possible role of the herbivores’ natural enemies, an important factor that can partly explain the observed pattern.

The theoretical framework referred to in Adams & Zhang (2009) focuses on plant–herbivore interactions and the evolution of plant defences. The third trophic level is mentioned but without reflecting on how temperature might influence predators, parasitoids or diseases. To not consider the influence of higher trophic level is unexpected, especially as the link between plants, herbivores and natural enemies has long been recognized (Lawton & McNeill 1979; Price *et al.* 1980). It is likely that different trophic levels respond differently to the same climate change (Voigt *et al.* 2003). In some cases, there is evidence indicating that plants respond more than herbivores and that the response to temperature becomes more indirect further up in the food chain (Forchhammer *et al.* 2008). However, these patterns have been found in studies of mammals and birds. When we consider insects, as is the case here, and in the study Adams & Zhang (2009), a different pattern emerges: plants respond least, (insect) herbivores intermediately and natural enemies the most to the same temperature change (Dunn 1952; Campbell *et al.* 1974; Virtanen & Neuvonen 1999; van Nouhuys & Lei 2004; ). Acknowledging these findings, we have recently developed a conceptual model where the anticipated response of all three trophic levels to climate change is presented (Berggren *et al.* 2009). When viewing the observations by Adams & Zhang (2009) from this perspective, their findings can at least partly be explained from a higher pressure from natural enemies on herbivores in warmer climates. However, the observed amount of folivory would depend on the temperature response of each species involved in the tritrophic interaction. In addition, regional climate may affect plant quality, which in turn affects the success of insect natural enemies (e.g. effects of immune competence, Haviola *et al.* 2007; Klemola *et al.* 2007).

To what extent there is a fundamental difference between different groups of herbivores and predators (mammals, birds and insects) and how they interact with plants and their response to temperature needs further investigation. Large-scale analyses including a vast array of taxonomical groups give a mixed picture. For example, Root *et al.* (2003) found that during the last decades non-tree plants showed a stronger phenological shift in spring than trees, whereas invertebrates and particularly birds showed a stronger phenological response than plants. Although broad-scale comparisons over many systems and taxonomical groups give important insights about trends and thus function as early warning signals (Walther *et al.* 2002), a more mechanistic understanding and better predictive ability is more likely to be achieved by in-depth studies of specific systems.

If we have the ambition to make predictions about how climate change might influence herbivore damage in the future, an understanding of the causes behind patterns of herbivory in relation to climate is crucial. We suggest that novel attempts to look for patterns in insect herbivory over climatic gradients should also include estimates of responses at the third trophic level. How this should be studied more explicitly will depend on the systems involved. Cage experiments could be used in many cases where the access of natural enemies to the cages is manipulated, to study the survival and performance of common insect herbivores that occur in the whole study area. This would give an estimate of plant quality effects and predator pressure in the area. The impact of different natural enemies (e.g. predators and parasitoids) can be separated with cleverly designed experiments that involve different types of cages. To use the cage method for estimating the relative contribution of bottom-up and top-down effects properly, one has to account for possible non-additive effects (Moreau *et al.* 2006). An alternative method is to experimentally study the disappearance rate of individuals in different introduced insects’ life-stages (eggs, larvae or pupae) in a range of study sites. However, these two methods mainly measure the pressure from predation and miss the effect of parasiotoids and disease. Despite this, predation pressure may be a good enough measure of interactions with a third trophic level, considering the fact that it has recently been recognized that the combined effect of several species of generalist predators may actually regulate the densities of many insect herbivores (Symondson, Sunderland & Greenstone 2002). To date, there are very few studies on how trophic interactions are affected by temperature in terrestrial habitats. A well-designed experiment – involving two plant types (herbs and grasses), one dominant herbivore (grasshopper) and two common predators (spiders) showed that experimental warming strengthened the effect of single predators (Barton & Schmitz 2009). Eventually, intraguild predation leads to the extinction of one of the spider species. A loss of predator diversity as a consequence of warming may have large consequences for the function and the stability of food webs.

Even more could be gained in our understanding of patterns of folivory if we knew the species involved. It would then be possible to study leaf damage at the edge of the distribution area of the plants, and from the colonizations and re-colonizations of herbivores, predators and parasitoids in these patches we would acquire a greater understanding of the dynamics of the different trophic levels. A carefully planned study would make it possible to unearth the mechanisms behind the observed insect folivory in different climates.

If theories on abiotic effects on biotic interactions of plant and insects were incorporated in studies on climate effects, we would achieve a greater understanding of how herbivores and their predators affect plant systems in a changing climate. We therefore emphasize the importance and benefits of greater collaboration between entomologists and plant ecologists. Such collaborations are becoming increasingly valuable as interactions between changes in climate and land-use patterns result in complex system responses.
